# Short curing time bulk fill composite systems: volumetric shrinkage, degree of conversion and Vickers hardness

**DOI:** 10.1590/1807-3107bor-2024.vol38.0030

**Published:** 2024-04-05

**Authors:** Camila Sobral SAMPAIO, João Luiz Bittencourt de ABREU, Batsheva KORNFELD, Eduardo Moreira da SILVA, Marcelo GIANNINI, Ronaldo HIRATA

**Affiliations:** (a) New York University, College of Dentistry, Advanced Clinical Fellowship in Aesthetics, Operative and Digital Dentistry, New York, NY, USA.; (b) Universidade Federal Fluminense – UFF, School of Dentistry, Analytical Laboratory of Restorative Biomaterials - LABiom-R, Niteroi, RJ, Brazil.; (c) New York University, College of Dentistry, Department of Biomaterials and Biomimetics, New York, NY, USA.; (d) Universidade Estadual de Campinas – Unicamp, Piracicaba Dental School, Department of Restorative Dentistry, Piracicaba, SP, Brazil.

**Keywords:** X-Ray Microtomography, Composite Resins, Hardness Tests

## Abstract

This study aimed to evaluate volumetric polymerization shrinkage, degree of conversion and Vickers hardness of four bulk-fill resin composites light-activated with their dedicated light curing units (LCUs). Four groups were evaluated, according to the type of composite and curing mode: Tetric EvoCeram Bulk-fill (TEBO) and Tetric EvoFlow Bulk-fill (TEBF) were light-activated with Bluephase Style 20i (20s, in high-mode), while Tetric Powerfill (TEPO) and Tetric Powerflow (TEPF) were light-activated with Bluephase PowerCure (3s). Volumetric polymerization shrinkage test (n = 6) was performed in standardized box-shaped class-I cavities of extracted third molars (4 x 4 x 4 mm). Teeth were scanned before and after resin composite application by micro-computed tomography, and acquired data were evaluated with Amira software. Degree of conversion (n = 5) was evaluated at the top and bottom surfaces of composite cylindric samples (4 mm diameter, 4 mm thickness) using an FT-IR spectrometer (spectra between 1,500 and 1,800 cm^-1^, 40 scans at a resolution of 4 cm^-1^). Three Vickers indentations (50 g / 15 s), spaced 500 μm apart, were performed on the top and bottom composite surfaces and averaged. One-way ANOVA was used for data evaluation. TEPF showed the lowest volumetric polymerization shrinkage (p < 0.05), while the other composites were not significantly different within each other (p > 0.05). All materials presented a significant decrease in degree of conversion and Vickers hardness when compared top to bottom surfaces (p < 0.05). Bottom to top surface ratios for degree of conversion ranged from 0.8 (TEBO and TEPO) to 0.9 (TEBF and TEPF), and from 0.4 (TEPO) to 0.7 (TEBF and TEPF) for hardness. In conclusion, resinous materials present a decrease in hardness and degree of conversion from top to bottom even when a higher power is used, while the flowable material TEPF showed the lowest volumetric shrinkage values compared to the other materials.

## Introduction

Resin composite restorations have been successfully incorporated into the daily practice of dental clinicians for more than 60 years, and undergone continuous efforts to improve its physical and mechanical properties, as well as decreasing technique complexity,^1^ Bulk-fill resin composites (BFRCs) were launched aiming for a new restorative concept, by allowing the placement of increments up to 4–5 mm with uniform polymerization, and thus reducing clinical time and simplification of the restorative procedure.^2-6^ This class of material is based on chemical composition modifications and more translucent formulations, relying on an alternative resin monomer, photoinitiators and different filler technologies.^3,4,7^ Even so, aiming for an even-faster procedure, manufacturers have launched higher radiant emittance light curing units (LCUs) and modified BFRCs for shortening the curing time, diminishing restorative procedure times.^8^ However, obtaining an acceptable hardness, usually within a bottom/top ratio threshold of equal or above 0.8, is paramount and may be a challenging task for these BFRCs.^3,5^ Different studies regarding BFRC materials report controversial results,^3^ and clinicians remain skeptic regarding their implementation in the clinical practice.

There are two classes of BFRCs, low filled or flowable, and highly filled with regular consistency materials. Flowable BFRCs are used as cavity bases under a regular consistency material,^9^ which can be a conventional resin composite or a BFRC. Regular consistency or packable materials have increased viscosity and do not require a covering layer, thus they can be used to fill the entire cavity and sculpt the occlusal surface simultaneously.^3^ Material viscosity seems to be an important factor on BFRCs curing success, as, typically, flowable materials have lower filler content and higher resin matrix content, as well as different types of monomers, which could result in higher volumetric polymerization shrinkage,^10^ although can also result in better depths of cure.^2^

High radiant emittance LCUs have been developed to polymerize new BFRCs in shorter periods of time and at a depth of approximately 4 mm,^8^ as well as the restorative materials have been modified to decrease shrinkage stress. The changes include a new stress reliever system specific for each material. For example, the use of prepolymerized particles with regular inorganic fillers (barium glass, silica), high filler, loading, increased translucency and new photoinitiator systems improve the polymerization process and influence shrinkage stress.^7,12,13^ The reduction of the photoactivation time is a concern since the low monomer conversion can alter the mechanical properties of the restorative materials.^14^ Regarding LCUs, a specific problem relates to the violet light, because it presents a more limited depth of penetration when compared to the blue light.^15^

The bottom part of the restorations is specifically affected by this situation, as increasing the thickness of the resin composite material might reduce the energy delivered to the bottom of it.^5^ During light-activation, the polymerization reaction starts rapidly after applying the irradiation source, which causes internal mobility restrictions within the growing polymer matrix network, which in turn causes reduction in the polymerization rate after few seconds.^16-19^ The polymerization reaction occurs in this rate mainly in the most superficial layers of the restoration, where the light reaches them with higher irradiance.^15^ However, in the deeper layers of the composite restoration less energy is available.^15^

At the beginning with the use of blue light for light-activation of composites, the recommended exposure time was 40 seconds. It has recently been reduced to 20 or 10 seconds and the consequences of this fast cure must always be evaluated in order not to compromise the performance of the restorative material. Investigations should also include fast-cure composites, which do not require longer irradiation times, because they contain alternative initiators with a tendency the produce a fast curing reaction with lower post-cure shrinkage.^3,7,17,20,21^ Several polymerization reaction analysis and their consequences have been suggested, however the main ones are related to the monomeric conversion, the mechanical properties and the behavior of the volumetric contraction produced by this reaction.^2-5,11,15,22-27^

The present study evaluated the volumetric polymerization shrinkage, degree of conversion and Vickers hardness of BFRCs light activated with different light curing times, using specific high radiant emittance LCUs. The null hypotheses tested were that different BFRCs would present the same volumetric polymerization shrinkage (1), and similar Vickers hardness (2) and degree of conversion (3) when increasing the depth of the composite restoration.

## Methodology

Tested materials and their compositions are provided in Table 1. Four different BFRCs were evaluated in this study: two light-activated for 20 seconds (Tetric EvoCeram Bulk Fill / TEBO and Tetric EvoFlow Bulk Fill / TEBF, Ivoclar Vivadent, Schaan, Liechtenstein), using a polywave LCU (Bluephase Style 20i, Ivoclar Vivadent) in high Power mode (1,200 mW/cm^2^radiant emittance) and two for 3 seconds (Tetric PowerFill / TEPO and Tetric PowerFlow / TEPF (Ivoclar Vivadent), using a high power LCU (Bluephase PowerCure, Ivoclar Vivadent) with radiant emittance of 3,050 mw/cm^2^.

**Table 1:** Studied materials compositions.

Material / Abbreviation	Composition
Tetric EvoCeram Bulk Fill / TEBO	The monomer matrix is composed of dimethacrylates (20-21 wt.%). The fillers contain barium glass, ytterbium trifluoride, mixed oxide and copolymers (79-81 wt.%). Additives, initiators, stabilizers and pigments are additional ingredients (<1.0 wt.%). The total content of inorganic fillers is 76–77% weight or 53–54% volume. The particle sizes of the inorganic fillers range between 40 nm and 3 μm.
Tetric EvoFlow Bulk Fill / TEBF	The monomer matrix is composed of dimethacrylates (28 wt.%). The fillers contain barium glass, ytterbium trifluoride and copolymers (71 wt.%). Additives, initiators, stabilizers and pigments are additional ingredients (<1.0 wt.%). The total content of inorganic fillers is 68.2 wt.% or 46.4 vol.%. The particle sizes of the inorganic fillers range between 0.1 μm and 30 μm.
Tetric PowerFill / TEPO	The monomer matrix is composed of dimethacrylates (20 – 21 wt%). The fillers contain barium glass, ytterbium trifluoride, mixed oxide and copolymers (79 – 80 wt.%). Additives, initiators, stabilizers and pigments are additional ingredients (< 1.0 wt%). The total content of inorganic fillers is 76 – 77 wt% or 53 – 54 vol%. The particle size of inorganic fillers is between 40 nm and 3 μm.
Tetric PowerFlow / TEPF	The monomer matrix is composed of dimethacrylates (28 wt%). The fillers include barium glass, ytterbium trifluoride and copolymers (71 wt%). Additional contents: additives, initiators, stabilizers and pigments (<1.0 wt%). The total content of inorganic fillers is 68.2 wt.% or 46.4 vol.% respectively. The particle sizes of the inorganic fillers range between 0.1 μm and 30 μm.
Adhese Universal VivaPen	Methacrylates, water, ethanol, highly dispersed silicon dioxide initiators and stabilizers
Bluephase Style 20i	20 seconds in high Power mode (1,200 mW/cm^2^ radiant emittance)
Bluephase PowerCure	3 seconds mode (3,050 mw/cm^2^ radiant emittance)

**Figure 1:**
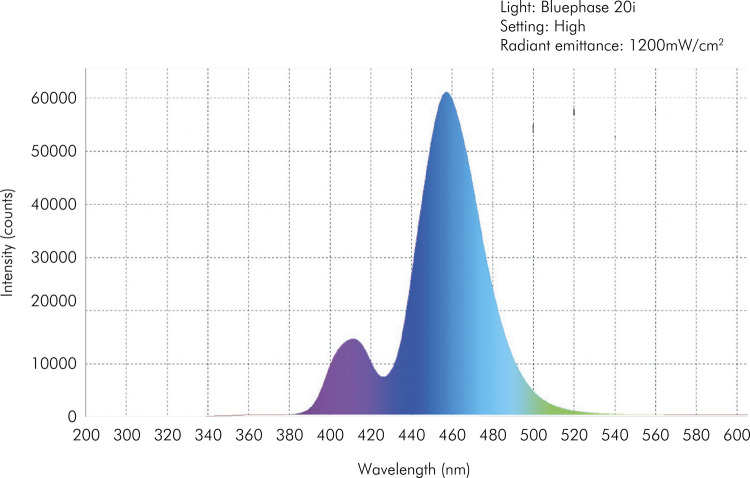
Characterization of Bluephase Style 20i LCU (Ivoclar Vivadent) using High Power mode. A peak at 410 and at 460 nm can be observed.

**Figure 2:**
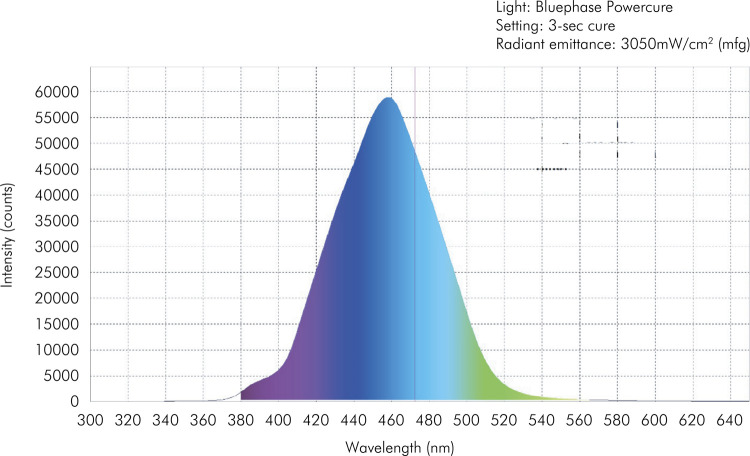
Characterization of Bluephase PowerCure LCU (Ivoclar Vivadent). Slightly broader spectrum (370 to 540nm), with a peak at 460nm can be observed.

Both LCUs were characterized by measuring the spectral irradiant profile, using a 6” integrating sphere (USS 060 SF, Labsphere Inc., Sutton, USA) containing a NIST-traceable light source, attached to a spectroradiometer (USB2000, Ocean Optics, Dunedin, USA), which provides output to computer software (Spectra Suite v5.1, Ocean Optics Inc., Dunedin, USA) (Figures 1 and 2).

### Volumetric polymerization shrinkage

**Figure 3:**
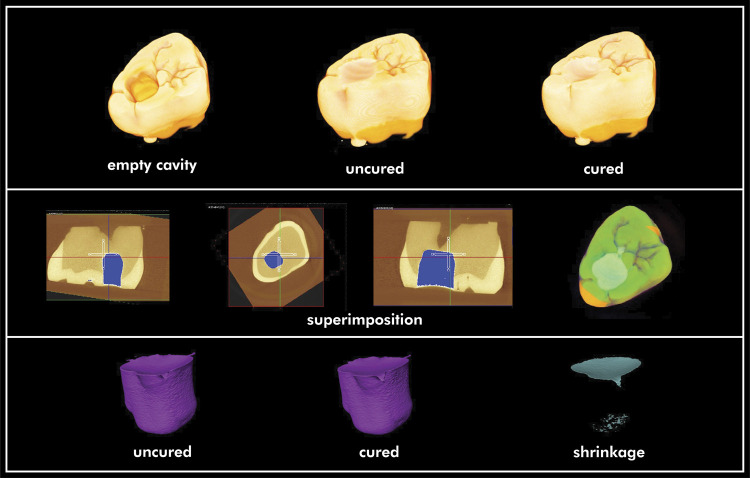
Step by step of the volumetric polymerization shrinkage test. Upper row, images of the empty tooth, tooth with uncured restoration and tooth with the cured restoration. Middle row, images of the superimposition steps, used to superimpose empty, uncured and cured scans. Lower row, images from the restoration digitally removed from the cavity: uncured, cured, and uncured minus cured resulting in the shrinkage volume.

Figure 3 depicts the steps of the volumetric polymerization shrinkage test. A standardized class I preparation (4 mm depth, 4 mm length and 4 mm width) was performed in twenty-four sound, freshly extracted human third molars, which were obtained according to protocols approved by the School of Dentistry Institutional Review Board (12/05). Cavity preparation were checked for dimensional accuracy with a digital caliper. Teeth were randomly divided into four groups, which represent the four composites tested (n = 6). Each tooth was scanned three times using a microcomputed tomography (µCT40, Scanco Medical, AG, Basserdorf, Switzerland), calibrated using a phantom standard at 70 Kvp/BH 200 mgHA/cm, with an operating condition of 70kVp–114 microamperes with a resolution giving 16 mm/slice, according to previous studies.^11,24,26^ The average of the total number of slices was approximately 250, and the average scan time was 28 minutes. The first microCT (mCT) scan was performed after cavity preparation for all teeth. Immediately after, bonding procedures were performed for all teeth with Adhese Universal VivaPen (Ivoclar Vivadent), light activated according to the same protocols determined for the BFRC from each group. After that, cavities were filled in bulk using their assigned resin composites, left uncured, and immediately placed inside the mCT holder. It is important to mention that clinically, flowable composites are not intended to be used in a single increment as they need an extra layer of a high-viscosity material. To avoid unwanted polymerization of the resin composite, the mCT holder was first covered with a dark plastic, avoiding contact with any light source, and then placed inside the mCT apparatus for the second scan and volume quantification. Subsequently, resin composites were light activated and inserted back into the holder for the third scan.

The mCT data were imported into a workstation and evaluated with Amira software (version 5.5.2, VSG, Burlington, USA). Superimposition of all three scan images was performed by the software, perfectly aligning them. Due to the similar radiodensity of the tooth and the resin composite, this procedure was performed in order to avoid scattering and possible noise formation. Registered mCT data of uncured and cured samples were subtracted from the cavity data, isolating the restoration (cavity filled with uncured resin composite minus cavity preparation, and cavity filled with cured resin composite minus cavity preparation), avoiding any scattering interference in the measurements. This procedure enabled both uncured and cured resin composite volumes to be isolated and quantified, allowing the volumetric polymerization shrinkage to be calculated as a percentage. Afterward, another subtraction was conducted for uncured minus the cured for imaging of the resin composite’s shrinkage. Data were analyzed using a one-way ANOVA. Tukey post hoc comparisons were carried out using 95% confidence intervals based on the pooled estimate of residual variability.

### Degree of conversion (%)

Bulk increments of each resin composite (n = 5) were inserted into a rubber mold (4.0 mm diameter and 4.0 mm thickness) positioned on the ATR crystal of the FT-IR spectrometer (Alpha-P/Platinum ATR Module, Bruker Optics GmbH, Ettilingen, Germany), and the spectra between 1,500 and 1,800 cm^-1^ were recorded with 40 scans at a resolution of 4 cm^-1^. Afterwards, the resin composite increments were covered with a polyester strip and a glass slide and light activated with the LCUs in close contact with the glass surface. The spectra at the top and bottom surfaces were recorded using the same FT-IR parameters, immediately one after the other to eliminate the influence of post-curing of any source in the obtained results. The degree of conversion was calculated from the ratio between the integrated area of absorption bands of the aliphatic C=C bond (1,638 cm^-1^) to that of aromatic C=C bond (1,608 cm^-1^), used as an internal standard, which were obtained from the cured and uncured increments, using the following equation:


$$DC\%=100x\left\{1-\frac{(\text{aliphatic}C=C)/(\text{ aromatic }C=C)\text{cured}}{(\text{aliphatic}C=C)/(\text{ aromatic }C=C)\text{ uncured }}\right\rfloor$$

Differences between groups were evaluated using a mixed model analysis in SPSS software, using a 95% confidence interval, and top and bottom values were compared for each resin composite.

### Microhardness

Vickers microhardness at top and bottom surfaces of the specimens used for the degree of conversion evaluation was performed for each resin composite. Before Vickers hardness measurements, all surfaces were wet polished (DPU 10, Struers, Denmark) with 2,500 and 4,000 grit SiC paper (250 rpm / 30 s in each paper). Three Vickers indentations (50 g / 15 s), spaced 500 μm apart were made in each surface (Micromet 5104 - Full MHT software, Buëhler, Lake Bluff, USA). The average of these three indentations was taking as the Vickers hardness (kgf/cm^2^) for each surface.

Differences between groups were evaluated using a mixed model analysis in SPSS software, using a 95% confidence interval, and top and bottom values were compared for each resin composite.

## Results

**Figure 4:**
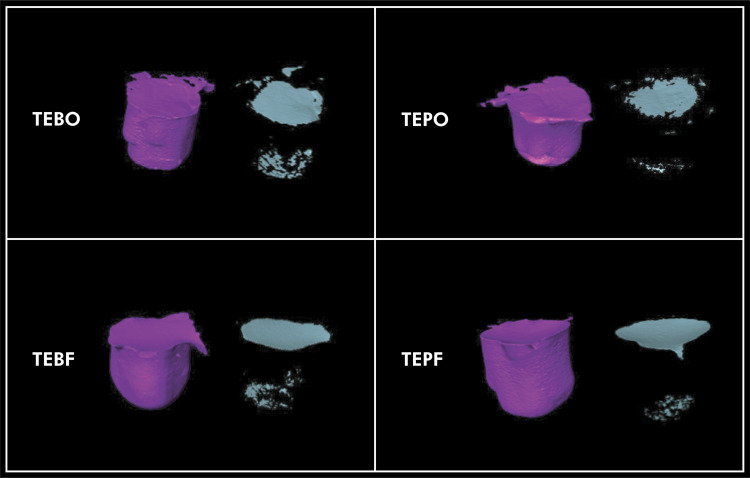
Volumetric polymerization shrinkage of each group. Restoration was digitally separated from the cavity surface. Left images present a cured restoration for each group (purple) and right images present the shrinkage of each restoration (green).

Table 2 shows the mean of volumetric polymerization shrinkage of composites. TEBO, TEBF and TEPF composites presented statistical higher volumetric polymerization shrinkage than that obtained for TEPO (p < 0.05) and did not differ among them (p > 0.05). Figure 4 shows representative images obtained from the µCT scans.

**Table 2:** Volumetric polymerization shrinkage means (stand. dev.) of each BFRC group.

Resin composite	Volumetric polymerization shrinkage (%)
TEBO	2.92 (1.11) A
TEBF	3.21 (0.47) A
TEPO	1.64 (0.75) B
TEPF	2.98 (0.13) A

Means followed by similar letters are not significantly different within groups (p > 0.05).

Table 3 shows means and standard errors of degree of conversion from the different groups for top and bottom locations. All materials presented a significant decrease in degree of conversion when compared top to bottom (p < 0.05). Bottom to top ratios ranged from 0.8 (TEBO and TEPO) to 0.9 (TEBF and TEPF). For both top and bottom location, TEPO showed the lowest values of DC%, statistically different than all other groups (p < 0.05). TEBF showed the highest DC% values for both top and bottom locations, although for top, it was statistically similar to TEBO (p = 0.549).

**Table 3:** Degree of conversion means (standard deviation) of each BFRC group, comparing top and bottom locations.

Resin composite	Degree of conversion (%)		Bottom-to-top ratio
	Top	Bottom	
TEBO	68.6 (3.42) Aa	53.3 (8.39) Bb	0.8
TEBF	69.6 (1.37) Aa	61.2 (1.25) Ab	0.9
TEPO	50.4 (3.31) Ca	39.1 (3.15) Cb	0.8
TEPF	58.0 (2.08) Ba	53.6 (2.88) Bb	0.9

Means followed by similar letters (upper case in columns, comparing BFRCs within the same location, and lower case in rows, comparing the same BFRC within different locations) are not significantly different (p > 0.05).

Table 4 presents the mean and standard errors from Vickers hardness from the different groups for top and bottom locations. Again, all materials also presented a significant decrease in Vickers hardness when compared top to bottom (p < 0.05). Bottom to top ratios ranged from 0.4 (TEPO) to 0.7 (TEBF and TEPF). For top location, the highest hardness results were observed for TEBO, followed by TEPO, both highly filled materials, statistically different within each other (p = 0.001). The lowest results were observed for the flowable material TEPF, which was also statistically different than TEBF (p = 0.024). When bottom location was evaluated, the highest values were observed for TEBF, while the other materials were not statistically different within each other (p > 0.05).

**Table 4:** Vickers Hardness means (stand. dev.) of each BFRC group, for top and bottom locations.

Resin composite	Vickers Hardness		Bottom-to-top ratio
	Top	Bottom	
TEBO	71.7 (7.12) Aa	40.9 (8.11) Ab	0.6
TEBF	44.5 (2.19) Ca	31.8 (2.74) Bb	0.7
TEPO	59.7 (5.43) Ba	25.8 (4.17) Bb	0.4
TEPF	36.7 (3.57) Da	25.2 (4.73) Bb	0.7

Means followed by similar letters (upper case in columns, comparing BFRCs within the same location, and lower case in rows, comparing the same BFRC within different locations) are not significantly different (p > 0.05).

## Discussion

Four BFRCs were selected for this study, all consisting of methacrylate-based monomers and presenting filler contents up to 71% for the flowable materials, and 79% for the highly filled materials. Volumetric polymerization shrinkage, degree of conversion and Vickers hardness were used to study the effect of fast light activation using high radiant emittance LCUs on different BFRCs. The three null hypotheses tested were rejected since the BFRCs studied resulted in statistical different values among all the aforementioned properties.

When choosing the appropriate resin composite for restoring teeth, particularly in the posterior region, a number of factors must be considered. The present study showed volumetric polymerization shrinkage values ranging from 1.64-3.21%, considered clinically acceptable and in accordance with previous studies.^11,24,26^ TEPO showed the lowest statistical values. TEPO was light polymerized for fewer seconds than TEBO (3s vs. 20s), thus, the lower volumetric polymerization shrinkage could be related to the lower degree of conversion obtained for this BFRC, observed in both top and bottom surfaces; a lower degree of conversion reflects in lower volumetric polymerization shrinkage, as they are directly related.^28^ Moreover, when TEPO is compared to the flowable BFRCs evaluated in the present study, the lower shrinkage can be explained by its higher amount of inorganic fillers, 79% against 71% from the flowable materials. A regular consistency resin composite commonly presents reduced monomer content, which may lead to less volumetric shrinkage.^10,11^ Since shrinkage occurs during monomer conversion to polymer, the lesser the filler content, the higher the resultant shrinkage when materials present similar composition, although it is not necessarily true when materials present different monomer compositions.^29^

The present study tested only BFRCs, with the objective to compare fast-cured BFRCs and their dedicated LCU with BFRCs that are light activated by the regular time (20 seconds), using a previous version of an LED LCU. The studies that compared BFRCs with conventional, incremental composites with the same consistency have shown that BFRCs present similar or lower volumetric polymerization shrinkage.^6,11,26^ Moreover, when packable and flowable materials are compared, authors frequently indicate flowable resin composites to be used as base filling, as it improves marginal integrity in dentin substrate, and use packable materials to substitute the enamel part of the restoration.^30,31^

Regarding degree of conversion analysis, the results of the present study indicated reduction with increased depth, in accordance with many investigations.^5,10,32^ However, it also needs to be emphasized that although a decrease from top to bottom was observed in all groups, all materials presented at least 50% of degree of conversion, except for TEPO at the bottom location that showed 39.1%. Other analysis must confirm if this degree of conversion is sufficient for clinical situations.^3^ However, a higher degree of conversion on top of the restoration might improve the wear and hydrolytic degradation resistance of the restoration at this location.^33^ It is also important to mention that the light received on the surface of the specimens is not homogeneously distributed, neither in irradiance nor in wavelength,^32^ and in the course of polymerization, it can promote a significant impact in the resin composite mass regarding both polymerization direction^15^ and degree of conversion.^32^ Both materials light polymerized for 20s (TEBO and TEBF) showed statistical increased DC% than the ones light polymerized for 3s (TEPO and TEPF) for top measurements, while for bottom measurements, both flowable materials (TEBF and TEPF) presented higher DC% than their counterparts in high consistency (TEBO and TEPO), and again, materials light polymerized for 20s showed statistical increased DC% compared to their counterparts light polymerized for 3s. Degree of conversion and translucency has been correlated in BFRCs,^3,7^ which can explain the higher DC% of the flowable materials compared to their counterparts in high consistency.

When Vickers hardness was evaluated, in agreement with degree of conversion results, all materials showed statistically significant decrease in their values from top to bottom. The increasing increment thicknesses tends to reduce the energy transferred to the bottom level of the samples, which explains the hardness and degree of conversion results.^5^ It has been showed that highly filled BFRCs exhibit higher indentation modulus and similar hardness as conventional nanohybrid resin composites,^7^ and superior than flowable materials, due to their greater inorganic content, explaining why both high consistency materials (in both 3 and 20s of light polymerization) presented superior microhardness than their flowable counterparts.

Regarding Vickers hardness, the minimum value suggested for an effective light activation procedure based on bottom to top hardness ratio is 0.8;^4,22^ however, no BFRCs evaluated in this study reached this ratio. Flowable materials were close, presenting a 0.7 ratio, while the packable BFRCs materials showed results farther from the minimum value. Although higher hardness values were not obtained, the present microhardness bottom results are numerically similar to those presented in several studies and even superior to several resin composites even in bottom location.^5,7,9^ However, limitations exist when comparing different studies, since different methodologies can be applied, thus, it is important to evaluate such decrease in bottom hardness property correlating it with clinical results.

LCUs used in this study emit blue and violet lights. The penetration of both lights inside the resin composite mass decrease significantly following the depth of restoration, being more critical for the violet light.^15^ Although the effect of using such high irradiance light-curing units is still not fully explored, a recent manuscript also observed that the high energy delivered in a short time via the wide-spectrum LCU was sufficient to produce an adequate DC, due to the materials’ ability to absorb energy from both types of wavelengths (violet and blue spectra) and its high reactivity to the lower wavelength,^34^ going in accordance to our results.

The results of the present study found a direct relation between degree of conversion and microhardness, and all materials showed a decrease in their properties from top to bottom; volumetric shrinkage varied among the different BFRCs. However, all investigated properties remained in the acceptable values from a clinical perspective, and thus can be indicated for restorative procedures. Recent data also supported the use of these Powercure materials in clinical applications after exhibiting good viscoelastic stability, performing comparably to conventional resin composites, but with only half the total energy delivery.^35^ Curing in less time with higher power did not improve the characteristics of the materials when compared to conventional curing times, however it did present acceptable results, thus can be seen as a real improvement of the technique, however, manufacturers need to continue evolving so it achieves better results than the conventional curing times and power. Although no ideal material exists until now, manufacturer’s improvements have allowed for simplification of clinical procedures with acceptable results.

## Conclusion

Based on the results obtained and limitations imposed by the present study, the following conclusions can be made:

- Volumetric polymerization shrinkage results of all studied BFRCs are within the acceptable range for resinous materials and the regular consistency BFRC Tetric Powerfill showed lower volumetric polymerization shrinkage results.
- Degree of conversion presented a decrease from top to bottom for all materials. The lowest reductions were around 22% for the two packable BFRCs. The BFRCs light polymerized for 20s presented increased DC% than the ones polymerized for 3s, when flowable materials and their counterpart in high consistency were compared.
- Vickers hardness presented a decrease from top to bottom for all materials. Bottom to top ratios ranged from 0.4 (Tetric PowerFill) to 0.7 (Tetric EvoCeram Bulk Fill and Tetric PowerFlow). For top location, the BFRCs light polymerized for 20s presented increased DC% than their counterparts polymerized for 3s, while high consistency materials presented increased values than their flowable counterparts. For bottom location, TEBO showed the highest results.
